# High Glucose-Mediated STAT3 Activation in Endometrial Cancer Is Inhibited by Metformin: Therapeutic Implications for Endometrial Cancer

**DOI:** 10.1371/journal.pone.0170318

**Published:** 2017-01-23

**Authors:** John J. Wallbillich, Srirama Josyula, Uksha Saini, Roman A. Zingarelli, Kalpana Deepa Priya Dorayappan, Maria K. Riley, Ross A. Wanner, David E. Cohn, Karuppaiyah Selvendiran

**Affiliations:** Division of Gynecologic Oncology, Comprehensive Cancer Center, The Ohio State University Wexner Medical Center, Columbus, Ohio, United States of America; University of South Alabama Mitchell Cancer Institute, UNITED STATES

## Abstract

**Objectives:**

STAT3 is over-expressed in endometrial cancer, and diabetes is a risk factor for the development of type 1 endometrial cancer. We therefore investigated whether glucose concentrations influence STAT3 expression in type 1 endometrial cancer, and whether such STAT3 expression might be inhibited by metformin.

**Methods:**

In Ishikawa (grade 1) endometrial cancer cells subjected to media with low, normal, or high concentrations of glucose, expression of STAT3 and its target proteins was evaluated by real-time quantitative PCR (qPCR). Ishikawa cells were treated with metformin and assessed with cell proliferation, survival, migration, and ubiquitin assays, as well as Western blot and qPCR. Expression of apoptosis proteins was evaluated with Western blot in Ishikawa cells transfected with a STAT3 overexpression plasmid and treated with metformin. A xenograft tumor model was used for studying the *in vivo* efficacy of metformin.

**Results:**

Expression of STAT3 and its target proteins was increased in Ishikawa cells cultured in high glucose media. *In vitro*, metformin inhibited cell proliferation, survival and migration but induced apoptosis. Metformin reduced expression levels of pSTAT3 ser727, total STAT3, and its associated cell survival and anti-apoptotic proteins. Additionally, metformin treatment was associated with increased degradation of pSTAT3 ser727. No change in apoptotic protein expression was noticed with STAT3 overexpression in Ishikawa cells. *In vivo*, metformin treatment led to a decrease in tumor weight as well as reductions of STAT3, pSTAT3 ser727, its target proteins.

**Conclusions:**

These results suggest that STAT3 expression in type 1 endometrial cancer is stimulated by a high glucose environment and inhibited by metformin.

## Introduction

Endometrial cancer is the most common gynecologic cancer and the fourth-most common cancer among women in the United States. 60,050 new cases of endometrial cancer and 10,470 deaths due to the disease are estimated for 2016. The incidence of endometrial cancer has been rising, with a 2.3% annual increase reported from 2006–2012 [1]. This increase has been attributed to the most common subtype of endometrial cancer, type 1 [2]. Type 1 endometrial cancer is comprised of grades 1 and 2 endometrioid adenocarcinoma [3]. Risk factors for type 1 endometrial cancer include obesity and diabetes, which have been increasing in prevalence in the United States[4, 5]. Further, in women with endometrial cancer, obesity leads to a 6.25 fold increased risk of dying due to the disease [6].

Regarding diabetes, while the disease is frequently co-morbid with obesity, it has been shown to independently increase the risk of endometrial cancer [7, 8]. Insulin resistance, insulin-like growth factors, hyperinsulinemia, and hyperglycemia have been implicated as potential etiologic agents in diabetes-associated endometrial cancer. However, existing data is conflicted as to the exact role, if any, of such diabetes-associated factors in the pathogenesis of endometrial cancer [9–14]. Given that STAT3 is over-expressed in endometrial cancer [15] and hyperglycemia has been shown to activate the STAT3 pathway in another disease site [16], we investigated whether high glucose concentrations might have an impact on STAT3 in endometrial cancer. Based on our pilot data that showed a high glucose-mediated increase in STAT3 in grade 1 endometrial cancer cells, we aimed to evaluate a potential inhibitor of such STAT3 activation.

Metformin, a medication commonly used in the treatment of type II diabetes, has gained recent traction as potential therapeutic agent for endometrial cancer [17, 18]. Epidemiologic studies have shown that metformin may lower risk of cancer and cancer-related death among patients with diabetes [19–21]. *In vitro* data has shown that metformin can inhibit cell proliferation in endometrial cancer cell lines [22, 23]. Based on these data, a phase II/III study is currently underway to evaluate metformin vs. placebo in patients receiving paclitaxel and carboplatin advanced or recurrent endometrial cancer (NCT02065687). Although metformin has been found to inhibit PI3K/AKT/mTOR and K-Ras signaling in endometrial cancer, the precise mechanism of the drug’s action against the disease has not been fully delineated [22, 24]. While the influence of metformin on STAT3 in endometrial cancer is unknown, metformin has been shown to inhibit STAT3-mediated cell proliferation in breast, esophageal, and bladder cancers [25–27]. Based on these reports as well as our preliminary results, we investigated whether metformin impacts high glucose-mediated STAT3 expression in type 1 endometrial cancer.

## Materials and Methods

### Materials

Metformin was purchased from Sigma-Aldrich (St. Louis, MO, USA). Sterile water was used as control (vehicle). Cell-culture medium (DMEM), fetal bovine serum (FBS), antibiotics, sodium pyruvate, trypsin, and phosphate-buffered saline (PBS) were purchased from Gibco (Thermo Fisher Scientific, Waltham, MA, USA). Polyvinylidene fluoride (PVDF) membrane and molecular-weight markers were obtained from Bio-Rad (Hercules, CA, USA). Antibodies against pNF-κB (p65), B-actin, cleaved caspases 3, 7, 8, and 9, cleaved PARP, Bcl-XL, fatty acid synthetase (FAS), Akt, and pAkt were purchased from Cell Signaling Technology (Beverly, MA, USA). Antibodies specific for STAT3, pSTAT3 ser727, Jak2, Cdk5, Bcl-2, cyclin D1, cyclin D2, survivin, MMP2, MMP9, and ubiquitin were purchased from Santa Cruz Biotechnology (Dallas, TX, USA). C-MYC was purchased from Abcam (Cambridge, MA, USA). Enhanced chemiluminescence (ECL) reagents were obtained from Amersham Pharmacia Biotech (GE Healthcare Bio-Sciences, Marlborough, MA, USA). All other reagents, of analytical grade or higher, were purchased from Sigma-Aldrich.

### Methods

#### Cell lines and cultures

The Ishikawa (grade 1) endometrial cancer cell line was used in this study (Obtained from ATCC). The cells were grown in DMEM with either low (1 mM), normal (5 mM), or high (25 mM) concentrations of glucose. The media was supplemented with 10% heat-inactivated FBS, 2% sodium pyruvate, 1% penicillin, and 1% streptomycin. Cells were grown in a 75 mm flask to 70% confluence at 37°C in an environment of 5% CO_2_ and 95% air. Cells were trypsinized (0.05% trypsin/EDTA) routinely.

#### Western blotting

Ishikawa endometrial cancer cells were incubated in their respective media. Cells were then incubated in high glucose DMEM treated with control (water), 10 mM metformin, or 20 mM metformin for 48h. Cell lysates were prepared in non-denaturing lysis buffer. The protein concentration in the lysates was determined using a Pierce detergent-compatible protein assay kit. For Western blotting, 30 to 60 μg of protein lysate per sample was denatured in 2X sample buffer and subjected to SDS-PAGE on a 4%-15% Tris–glycine gel. The separated proteins were transferred to a PVDF membrane and then blocked with 5% nonfat milk powder (w/v) in TBST (10 mM Tris, 100 mM NaCl, 0.1% Tween 20) for 1 h at room temperature or overnight at 4°C. The membranes were incubated with the primary antibodies described above. The bound antibodies were detected with horseradish peroxidase (HRP)-labeled sheep anti-mouse IgG or HRP-labeled donkey anti-rabbit IgG (Amersham) using an enhanced chemiluminescence detection system (ECL Advanced kit).

#### RNA isolation and Reverse Transcription PCR (RT-PCR)

Total RNA was isolated from samples using the RNeasy Mini Kit (Qiagen, Valencia, CA, USA). RNA samples with an optical density A260/A280 ratio between 1.8 and 2.1 were used. RT‐PCR was then performed using the Transcriptor First Strand Complementary DNA (cDNA) Synthesis Kit (Roche Diagnostics, Indianapolis, IN, USA) to synthesize cDNA. RT‐PCR was performed with 1mg of RNA template. The reaction was carried out using the Veriti Thermal Cycler (Applied Biosystems, Thermo Fisher Scientific) and random hexamer primers.

#### RNA Quantitative Real Time PCR (qPCR)

The genes studied for their relative genetic expression patterns are provided in “S1 Table”. LightCycler 480 SYBR Green I Master Mix (Roche Diagnostics) was used to analyze 100 ng of cDNA from each experimental condition along with their respective primers. Quantitative real-time PCR (qPCR) was performed using the StepOnePlus Real-Time PCR System (Applied Biosystems). Data were analyzed using comparative CT method with internal control GAPDH levels to normalize differences in sample loading. Results (mean ± standard deviation of triplicate reaction wells) represent the n-fold difference of mRNA transcript levels in a particular sample compared with samples of control Ishikawa cells.

#### MicroRNA isolation and RT-PCR

At the conclusion of the experimental protocol, microRNA was isolated using mirPremier microRNA Isolation Kit (Sigma Aldrich). MicroRNA samples with an optical density A260/A280 ratio between 1.8 and 2.1 were used for RT-PCR. Poly-A Tail addition and cDNA synthesis was performed using the MystiCq microRNA cDNA Synthesis Mix (Sigma Aldrich). The reactions were carried out with 200ng of microRNA template using the Veriti Thermal Cycler.

#### MicroRNA qPCR

The genes studied for their relative genetic expression patterns are provided in S1 Table. MystiCq microRNA SYBR Green qPCR MasterMix (Sigma Aldrich) was used to analyze 100 ng of cDNA from each experimental condition with MystiCq microRNA qPCR Assay primers and MystiCq Universal PCR primer (Sigma Aldrich). Quantitative real time PCR was performed using the StepOnePlus Real-Time PCR System. Data were analyzed using comparative CT method with internal control SNORD44 levels to normalize differences in sample loading. Results (mean ± standard deviation of triplicate reaction wells) represent the n-fold difference of microRNA transcript levels in a particular sample compared with samples of control Ishikawa cells.

#### Cell viability by SRB assay

Cell viability was determined by a colorimetric assay using Sulforhodamine B (SRB). SRB binds to protein components of cells that have been fixed to tissue-culture plates by trichloroacetic acid (TCA). SRB is a bright-pink aminoxanthene dye with two sulfonic groups that bind to basic amino-acid residues under mild acidic conditions, and dissociate under basic conditions. As the binding of SRB is stoichiometric, the amount of dye extracted from stained cells is directly proportional to the cell mass. cancer cells were grown to 80% confluence in 100 mm culture plates, trypsinized, counted, and seeded in 96-well plates with an average population of 8,000 cells/well and collected for the cell viability assays at 24 and 48 hours post metformin treatments ranging from 0.5 to 20 mM. All experiments were done using six replicates and repeated at least three times.

#### Cell migration assay

Cell migration assay was done using the wound-healing method [28, 29]. Cells were plated at equal density and grown to 90% confluence. Wounds were created using a sterile pipette tip. Cells were then rinsed with medium and replaced with fresh medium and incubated with either control, 10 mM metformin, or 20 mM metformin for 24h. Areas of wound were photographed at various time points with a phase-contrast microscope. Areas of absent wound closure were quantified with ImageJ (National Institutes of Health, Bethesda, MD, USA).

#### Ubiquitin assay

Cells were cultured with 25 μM of the proteasome inhibitor MG132 (Calbiochem, EMD Millipore, Billerica, MA, USA) while under treatment with metformin. Cell lysates were incubated with primary antibody and immunoprecipitated with magnetic beads. The immunoprecipitate was analyzed via Western blot, probing for ubiquitin.

#### STAT3 overexpression

The STAT3 overexpression experiments were performed using a wild-type STAT3 plasmid. The plasmid was transfected into Ishikawa endometrial cancer cells using the Avalanche transfection reagent (EZ Biosystems, College Park, MD, USA) according to the manufacturer's protocol. 24h after the transfection of the STAT3 gene, control or metformin 20 mM was added and incubated for 48h. The cells were then subjected to Western blot analysis.

#### Animal model study: Endometrial tumor xenografts in mice

The Institutional Animal Care and Use Committee (IACUC) approved this study. The 15 mice were observed regularly for any signs of distress like anxiety, loss of appetite, pain in the site of injection etc. post injection with the cancer cells, using clinical observation chartsheets. Any pain signs were managed by administering buprenorphine until the pain diminishes. Once the tumor started growing, if it was observed that the tumor site was bleeding/ulcerating, or growing vigorously making it impossible for the mouse to move/eat/drink, we euthanised the mice using CO2 exposure followed by cervical dislocation. In case we observed that any of the mice had hunched posture, altered feeding habits, provoked behavior, piloerection etc, we consulted our veterinary physicians and technicians for their expert advice on what steps should be taken to address the issues on the mice.

Cultured Ishikawa cancer cells (1 × 10^6^ cells in 100 of PBS) were subcutaneously injected into the flank of 6-week-old BALB/c nude mice from the Ohio State University nude mice core lab. After tumors were noted to be 3–5 mm in diameter, treatment was initiated. The groups were treated with either control (Teklad LM-485 Mouse/Rat Sterilizable Diet), 100 mg/kg, or 200 mg/kg metformin supplemeted by driniking water. The treatment groups received metformin dissolved in their water source. After 4 weeks of treatment, the mice were sacrificed and the tumors were resected. The tumor tissues were then weighed and subjected to Western blot and RT qPCR analyses.

#### Statistical analysis

Results were expressed as mean ± S.D. Comparisons between groups were made by a Student's t-test. The significance level was set at p ≤ 0.05.

## Results

### High glucose concentrations increase expression of STAT 3 and its target proteins in grade 1 endometrial cancer cells

**Fig 1:**
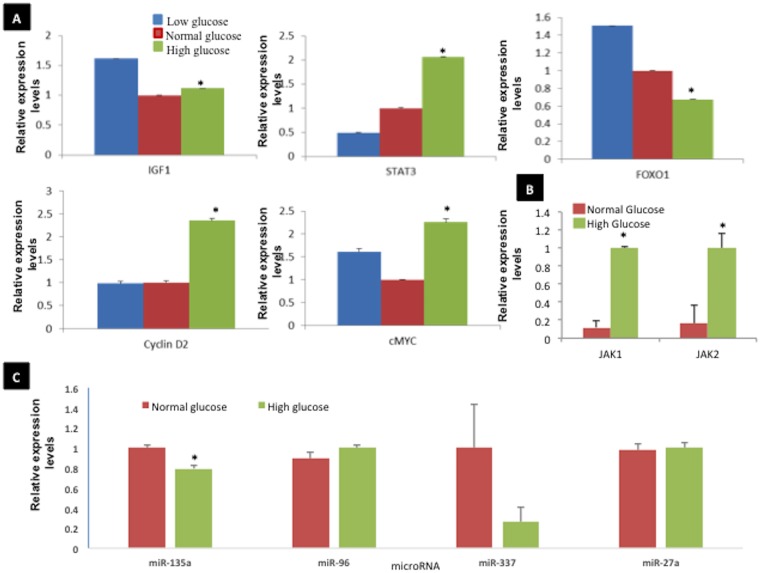
Relative expression of STAT3 in grade 1 endometrial cancer cells with respect to glucose concentration. **A & B**. Ishikawa cells were cultured in low, normal, or high glucose media for 24 hours. Relative expression of STAT3 and its regulatory genes were calculated by normalizing the values of normal glucose to 1. **C**. STAT3-associated microRNA was then evaluated with qPCR. Groups significantly different than control (p<0.05) are indicated with an asterisk (*).

Ishikawa endometrial cancer cells were cultured in low, normal, or high glucose media for 24 hours; RNA was extracted from the various treatment groups, reverse transcribed, and subjected to gene-specific real time quantitative PCR (qPCR). The mRNA expression of oncogenic proteins STAT3, IGF1, cyclin D2, and c-MYC increased with respect to increasing glucose concentrations while FOXO1 (tumor suppressor) showed decreased mRNA expression (Fig 1A). In addition, high glucose increased mRNA expression of STAT3 upstream regulators (Jak1 and Jak2, Fig 1B). Expression of miR-135a, a tumor suppressor associated with STAT3, was inhibited by a high glucose environment (Fig 1C).

### Metformin reduces high glucose-mediated expression of STAT3 and its regulatory proteins in endometrial cancer cells

**Fig 2:**
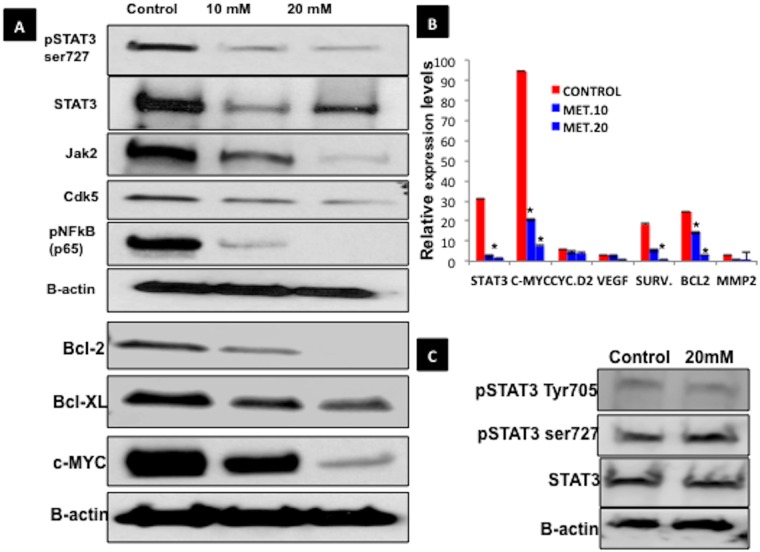
Metformin inhibits STAT3, its regulatory proteins and upregulated apoptosis-related proteins, in grade 1 endometrial cancer cells. **A**. Western blot of STAT3 and its regulatory proteins in Ishikawa cells after treatment with control, 10 mM, or 20 mM metformin for 48h in high-glucose conditions. **B**. qPCR of STAT3 and some of its regulatory genes in Ishikawa cells after treatment with control, 10 mM, or 20 mM metformin for 48h. Groups significantly different than control (p<0.05) are indicated with an asterisk (*). **C**. Metformin did not affects pSTAT3 and STAT3 in normal glucose conditions.

To evaluate the impact of metformin on the STAT3 pathway in grade 1 Ishikawa endometrial cancer cells in high glucose conditions, protein and mRNA expression studies were performed. Western blot demonstrated dose-dependent inhibition of expression of pSTAT3 ser727, total STAT3, and STAT3 regulatory proteins Jak2, Cdk5, and pNFkB (p65) (Fig 2A). Metformin also decreased STAT3 downstream target genes c-MYC, Bcl-2 and Bcl-xL, which suggests alterations in pro-proliferative and anti-apoptotic function (Fig 2A). Further analysis with qPCR showed that metformin treatment led to significant inhibition of STAT3 and other associated genes such as c-MYC, Bcl-2, survivin and VEGF (Fig 2B). Our results demonstrate that metformin targets STAT3 and its associated genes. In addition, we have showed that whether the metformin affects pSTAT3 expression in normal glucose conditions and it seems that pSTAT3 is not significantly elevated in normal glucose conditions although the TSTAT3 is expressed (Fig 2C). Moreover metformin treatment seems to have significant effect on the total STAT3 expression as well but is affected in Ishikawa cells grown in high glucose medium.

### Metformin increases degradation of pSTAT3 ser727 in endometrial cancer cells

**Fig 3:**
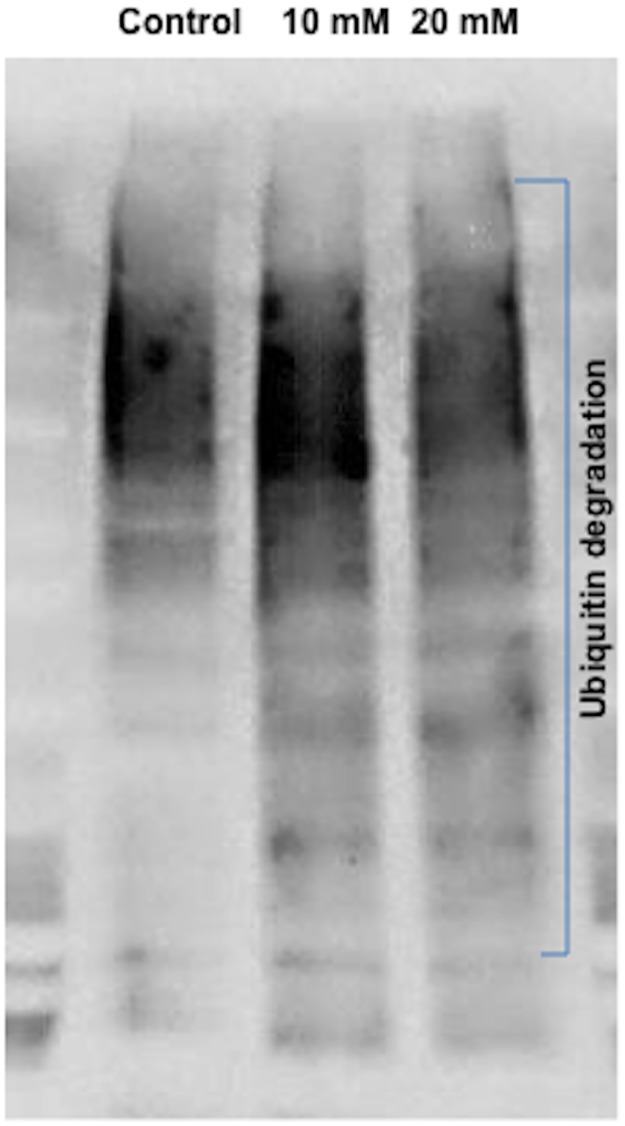
Degradation of pSTAT3 ser727 in grade 1 endometrial cancer cells treated with meformin. After Ishikawa cells were treated with control, 10 mM, or 20 mM metformin in high glucose media for 48h, samples were subjected to ubiquitin assay. The ubiquitinated proteins were subjected to immunoblot for pSTAT3 Ser727 and blotted with ubiquitin antibody. A ubiquitination smear of pSTAT3 Ser727 is seen in the metformin treated ishikawa cells under proteasomal inhibition using MG-132.

To examine the direct effect of metformin on activated STAT3, samples from Ishikawa cells treated with metformin (vs. control) for 48h were subjected to ubiquitin assay. An increased degradation of pSTAT3 Ser727 was observed in the metformin treatment groups (Fig 3).

### Effect of metformin on proliferation, survival, migration, and apoptosis of endometrial cancer cells

**Fig 4:**
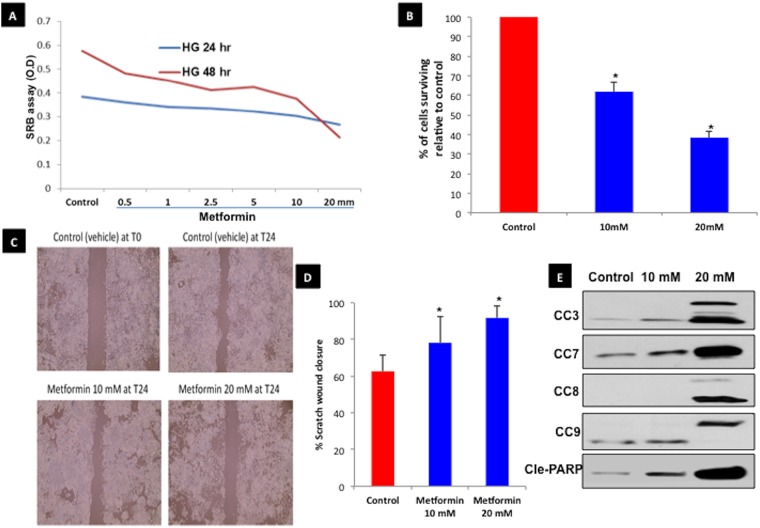
Metformin inhibits grade 1 endometrial cancer cell proliferation, survival, migration, and induces apoptosis. **A**. Sulforhodamine B (SRB) assay measured Ishikawa cell proliferation with increasing concentrations of metformin after 24h or 48h, in high-glucose media. **B**. Proportion of Ishikawa cells surviving (compared to control) after treatment with 10 mM or 20 mM of metformin. **C**. Cell migration assay, with Ishikawa cells subjected to control, 10 mM, or 20 mM metformin for 24h. Control (0h) is also shown. **D**. Quantification of % wound closure; the 10 mM and 20 mM treatment groups were each significantly different than control (p<0.05; noted with asterisk [*]). **E**. Western blot of apoptosis-related protein expression in Ishikawa cells after treatment with control, 10 mM, or 20 mM for 48h.

Using an SRB assay, we found that Ishikawa cancer cell proliferation decreased with increasing concentrations of metformin after 24h or 48h in high glucose media (Fig 4A). Metformin inhibited Ishikawa cell survival, with the least cell survival observed after treatment with the highest dose (20 mM) of the drug (Fig 4B). In a cell migration assay, metformin (at 10 mM and 20 mM) significantly inhibited scratch wound healing relative to control after 24h of treatment (Fig 4C and 4D). Additionally, western blot showed dose-dependent increases in apoptotic proteins (cleaved caspases 3, 7, 8, 9 and cleaved PARP) in metformin treatment groups (Fig 4E).

### The impact of metformin on xenograft endometrial tumor and expression of STAT3 and its target proteins in vivo

**Fig 5:**
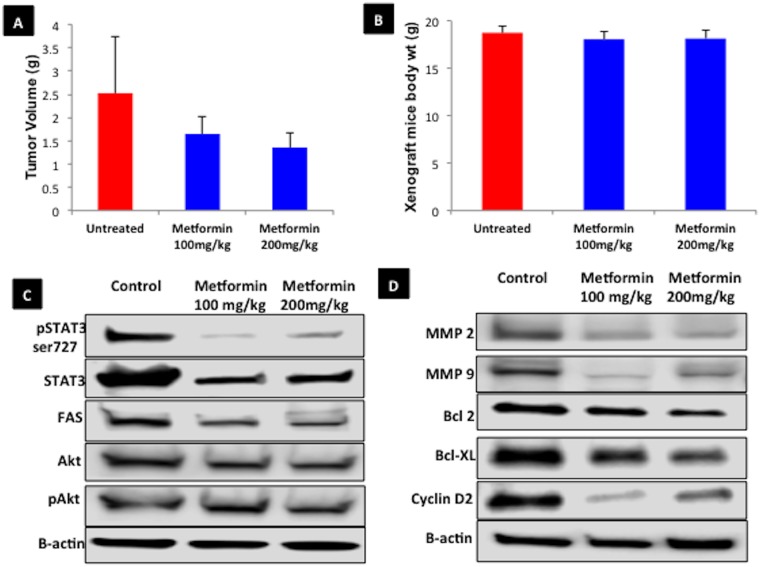
Xenograft endometrial tumor weight and expression of STAT3 in mice treated with metformin. A xenograft study was done in which nude mice were injected with 1 x 10^6^ Ishikawa endometrial cancer cells subcutaneously in the right flank. After tumors were at least 3–5 mm in diameter, treatment with control, metformin 100 mg/kg, or metformin 200 mg/kg was started. Mice were sacrificed after 4 weeks of treatment. **A**. Tumor weight (in grams) from the mice with the 3 largest tumors in each group. **B**. Average body weight (g) of the mice in each group at conclusion of the study. **C**. Western blot results of STAT3 and associated proteins after treatment with control, 100 mg/kg, or 200 mg/kg of metformin.

Xenograft mice injected with Ishikawa endometrial cancer cells were either untreated (control) or treated with metformin (100 and 200mg/kg). The mice were euthanized after 4 weeks and the tumor weight was measured in each group. There was a trend toward decreasing tumor weights with increasing doses of metformin, but the differences were not statistically significant (Fig 5A). The total body weight of the mice at conclusion of the experiment was similar among the treatment groups (Fig 5B), suggesting that metformin–treated animals did not show any gross signs of toxicity based on the body weight profile. Collected the tumor tissues were subjected to western blot showed decreased protein expression of pSTAT3 ser727, total STAT3, Bcl-2, Bcl-XL, FAS, cyclin D2, MMP2, and MMP9 in metformin treated vs. control mice. Akt and pAkt protein expression did not change with respect to treatment (Fig 5C). Although the metformin treatments inhibited expression of STAT3 and its target proteins, the change in tumor weight was not significant in the 100 and 200 mg/kg metformin treatment groups. We are going to analyze the metformin in vivo efficacy in our future experiments with different metformin doses and treatment periods.

### The influence of metformin on apoptosis proteins in endometrial cancer cells overexpressing STAT3

**Fig 6:**
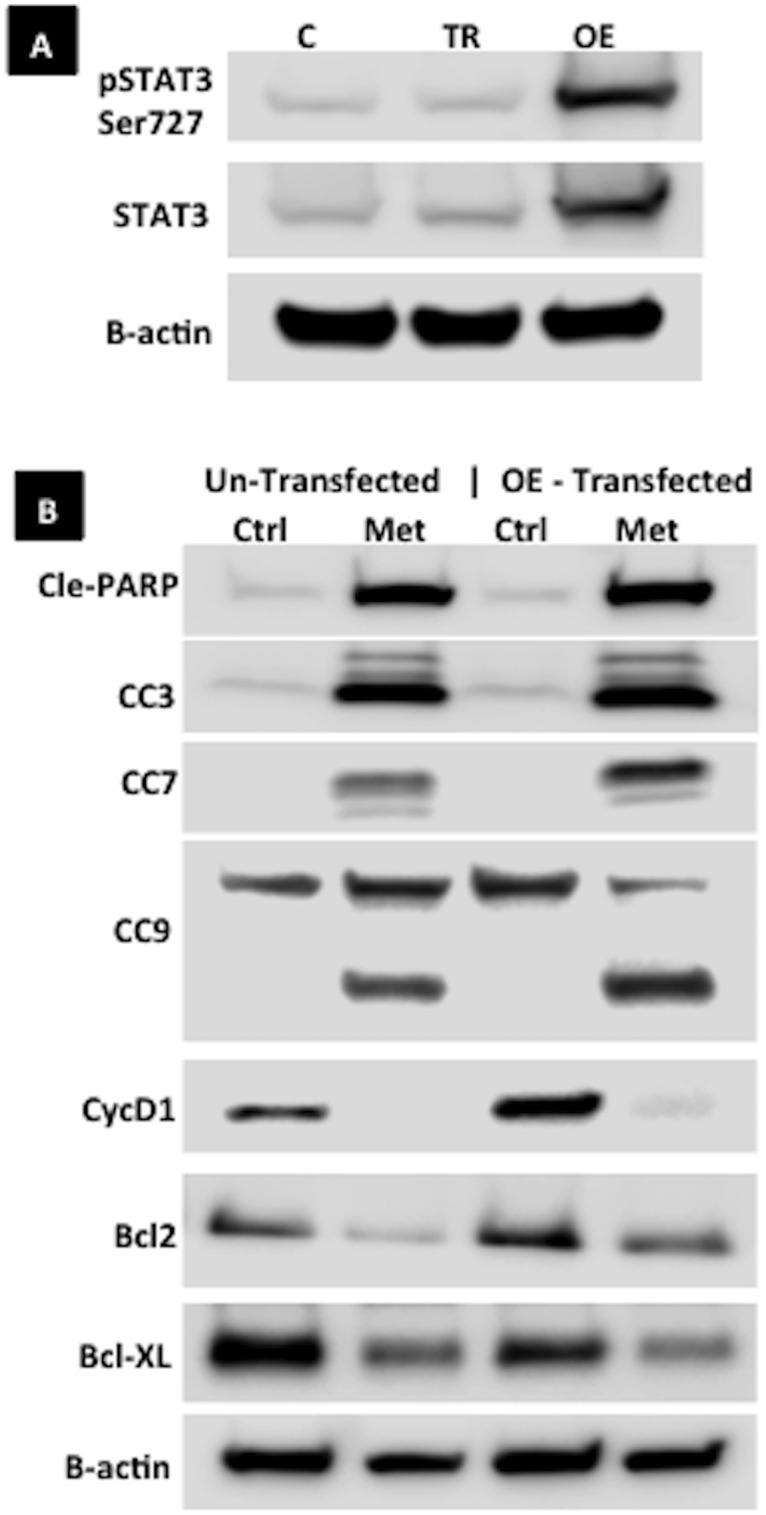
Expression of apoptosis and cell proliferation-related proteins in metformin-treated grade 1 endometrial cancer cells overexpressing STAT3. Ishikawa endometrial cancer cells were transfected with a STAT3-overexpressing plasmid. **A**. Western blot confirming overexpression of pSTAT3 ser727 and total STAT3. **B**. Western blot of proteins involved in apoptosis or cell proliferation in control or STAT3-overexpressing Ishikawa cells treated with control or 20 mM metformin in high-glucose medium for 48h. (C: control, TR: transfection reagent only, OE: transfected with STAT3-overexpressing plasmid, Ctrl: control, Met: metformin).

To investigate the effect of metformin on apoptosis-related proteins, Ishikawa endometrial cancer cells were transfected with a STAT3-overexpressing plasmid and treated with metformin. Overexpression of pSTAT3 ser727 and total STAT3 was confirmed prior to treatment (Fig 6A). After treatment with metformin, the protein expression of cleaved PARP, cleaved caspases 3, 7, 9, cyclin D1, Bcl-2, and Bcl-XL on Western blot for metformin (vs. control) was similar between the untransfected and STAT3-overexpressing groups was (Fig 6B). These results suggest that metformin promotes apoptosis and inhibits proliferation in grade 1 endometrial cancer cells through a mechanism not entirely dependent on STAT3 inhibition.

## Discussion

The present study found that high glucose concentrations increase expression of STAT3 and its target proteins in grade 1 endometrial cancer cells. We also observed that metformin affects grade 1 endometrial cancer cell growth, apoptosis induction, and inhibits STAT3 and its target proteins in both *in vitro* and *in vivo* (with a murine xenograft model).

Although it is known that the tumorigenesis of obesity-associated (type 1) endometrial cancer is linked with enhanced cellular glucose uptake [30, 31] and increased metabolism, the molecular pathway alterations for these processes have not been clearly delineated. This study helps contribute to the understanding of these mechanisms, showing that high glucose concentrations alter expression of STAT3 and its target proteins in type 1 endometrial cancer cells. We have previously reported that constitutive expression of pSTAT3 occurs in human endometrial tumor tissues and endometrial cancer cell lines [15]; other studies have shown that consistent STAT3 expression regulates the endometrial cancer growth and angiogenesis [32, 33].

While STAT3 is a well-established role player in multiple types of cancer [34], recent evidence indicates the importance of STAT3 signaling pathways in obesity-associated cancers and indirectly suggests that diabetes could enhance cancer development via STAT3 activation [35]. The present study supports this possibility, as high glucose treatment was found to up-regulate STAT3 and its target genes in type 1 endometrial cancer cells. Although prior research has shown that STAT3 can be targeted in various cancers using small molecular inhibitors and natural compounds, there is no clinically available STAT3 inhibitor. Given recent evidence showing that metformin targets STAT3 pathways in triple negative breast cancer cells, and that metformin is widely available and in common clinical use (for type 2 diabetes), metformin has the potential for rapid therapeutic implementation for STAT3-overexpressing cancers[25]. Further, metformin has been found to reduce the risk of cancer in diabetic patients and combination treatment of metformin with small molecule STAT3 inhibitors has been proposed as a method of improving therapeutic efficacy in breast cancer[36].

In the present study we show that metformin suppresses both pSTAT3 Ser727 and total STAT3 expression in a grade 1 endometrial cancer cell line cultured in high glucose media. We previously reported that pSTAT3 Ser727 is highly elevated in endometrial cancer cell lines and human samples compared to pSTAT3 Tyr705, which is the form of activated STAT3 more commonly found in STAT3-overexpressing cancers (including ovarian cancer) [15, 37]. Although metformin has been noted to act through alteration of AMPK/mTOR, K-Ras, and the eIF4E-binding protein in diabetic patients and cancer cells [22, 23, 38, 39], the precise mechanisms for its inhibition of endometrial cancer are not yet fully understood. This is first study to show that metformin targets pSTAT3 by degrading the protein and suppresses the upstream regulator JAK2 as well as the positive feedback regulator pNF-κB proteins in type 1 endometrial cancer cells. Further, we have found that metformin treatment decreases CDK5 in type 1 endometrial cancer cells. It has previously been shown that STAT3 is phosphorylated at a Ser727 residue by CDK5, a unique member of the CDK family [40, 41]. Our findings are in agreement with previous studies that found metformin treatment suppresses the development of prostate and melanoma tumors by targeting STAT3 and NF-κB in cancer stem-like cells during the early inflammatory process (known to be associated with carcinogenesis) [42–44]. In addition, targeting STAT3 can control abnormal metabolic conditions such as diabetes and obesity, which might reduce the risk of cancer associated with those conditions [34, 45].

The constitutive activation of STAT3 has been implicated in the regulation of cancer cell survival (survivin, Bcl-xL), proliferation (cyclin D1/D2) and anti-apoptosis (Bcl-2) [46]. Previous reports have shown that activation of STAT3 reduces the apoptotic effect of chemotherapeutic drugs in various cancer cells by up-regulating Bcl-2 and Bcl-xL [47]. Several studies have confirmed that STAT3 inhibition by small molecule inhibitors results in tumor cell apoptosis in both in vitro and in vivo [48, 49]. In this study, metformin inhibits cell survival, migration, induces apoptosis, and impedes tumor growth. Metformin significantly suppresses the STAT3 target gene Bcl-2 and increases multiple cleaved caspases as well as cleaved PARP, indicating an induction of apoptosis. These data are in agreement with previous studies that found metformin inhibits cancer cell proliferation and induces apoptosis through the inhibition of STAT3, ERK, and IGF1 pathways [25, 50]. In addition, we have shown that metformin-mediated induction of cell death might not completely depend on STAT3 inhibition in type 1 endometrial cancer cells. Additional studies are needed to explore these selective pathways or other pathways involved in induction of cell death with regard to metformin treatment in endometrial cancer.

In conclusion, we have demonstrated a relationship between a high-glucose environment and STAT3 activation in type 1 endometrial cancer cells. Further, through in vitro and in vivo investigations we have found that metformin inhibits cell proliferation, reduces cell survival, induces apoptosis, and inhibits STAT3 and its target proteins such as survivin, Bcl-2, Cyclin D1, and c-MYC. Metformin shows substantial possibilities for further development as a potential agent for treating type 1 endometrial cancer and other diabetes-associated cancers.

## Supporting Information

S1 Table Primer sequences for the genes used in the quantitative real time PCR studies. (PDF)
