# Supplementary material for: High Glucose-Mediated STAT3 Activation in Endometrial Cancer Is Inhibited by Metformin: Therapeutic Implications for Endometrial Cancer
Source: PLoS One. 2017 Jan 23;12(1):e0170318. doi: 10.1371/journal.pone.0170318 (PMC5256996; doi:10.1371/journal.pone.0170318)
Supplement: S1 Table — (PDF) [file pone.0170318.s001.pdf]

Supplemental Table 1. Primers used in RT-PCR and qPCR experiments.

Name of gene	Forward primer	Backward primer
GAPDH	AACGGGAAGCTTGTCATCAATGGAAA	GCATCAGCAGAGGGGGCAGAG
IGF1	TCGCATCTCTTCTATCTGGCCCTGT	GCAGTACATCTCCAGCCTCCTCAGA
STAT3	ATGGCCCAATGGAATCAGC	TTATTTCCAAACTGCATCAA
FOXO1	GCCATGTAAGTCCCATAGA	ATCGGAACAAGAACGTGGAATC
Cyclin D2	CTGTGTGCCACCGACTTTAAGTT	GATGGCTGCTCCCACACTTC
c-MYC	AATGAAAAGGCCCCCAAGGTAGTTATCC	GTCGTTTCCGCAACAAGTCCTCTTC
JAK1	CTCTGACGTCTGGTCTTTTGG	GTTGGGCCTATCATTTTCAGGAAC
JAK2	TGGAGCTTTGGAGTGGTTCTG	TGCCAATCATACGCATAAATTCC
VEGF	TGGGAACCGGAACCTCACTATC	GTCTTTTCCTGGGCACCTTCTATT
Survivin	AGAACTGGCCCTTCTTGGAGG	CTTTTTATGTTCCTCTATGGGGTC
Bcl-2	CTGCACCTGACGCCCTTCACC	CACATGACCCCACCGAACTCAAAGA
MMP2	TCTCCTGACATTGACCTTGGC	CAAGGTGCTGGCTGAGTAGATC
miR-135a	TATGGCTTTTTATTCCTATGTGA	TCACATAGGAATAAAAAGCCATA
miR-96	TTTGGCACTAGCACATTTTTGCT	AGCAAAAATGTGCTAGTGCCAAA
miR-337	CTCCTATATGATGCCTTTCTTC	GAAGAAAGGCATCATATAGGAG
miR-27a	TTCACAGTGGCTAAGTTCCGC	GCGGAACTTAGCCACTGTGAA
